# Evaluation of Multiple Tissue Levels Frequently Upstages Patients With Clinically Localized Thin Primary Cutaneous Melanoma

**DOI:** 10.1111/cup.14726

**Published:** 2024-10-02

**Authors:** Louise A. Jackett, James P. Gullifer, Richard A. Scolyer

**Affiliations:** ^1^ Department of Anatomical Pathology Peter MacCallum Cancer Centre Melbourne Victoria Australia; ^2^ Melanoma Institute Australia, The University of Sydney Sydney New South Wales Australia; ^3^ Tissue Pathology and Diagnostic Oncology Royal Prince Alfred Hospital & NSW Health Pathology Sydney New South Wales Australia; ^4^ Faculty of Medicine and Health The University of Sydney Sydney New South Wales Australia; ^5^ Charles Perkins Centre The University of Sydney Sydney New South Wales Australia

**Keywords:** Breslow, melanoma, pathology, staging, ulceration

## Abstract

**Background:**

Breslow thickness (BT), ulceration, and microsatellitosis are critical prognostic parameters for cutaneous melanoma staging. These parameters can vary depending on the number of tissue levels examined from individual paraffin blocks. We sought to evaluate all prognostic histopathologic parameters in melanoma for their variations between levels, taken at regular intervals, in a single study.

**Methods:**

We analyzed 40 consecutive cases of primary cutaneous (nonacral) melanoma through five hematoxylin and eosin sections, taken at 100 μm intervals, for staging and prognostic parameters.

**Results:**

Examination of additional levels resulted in (a) an increase in BT in 47.5% (19 out of 40) of cases and (b) detection of ulceration in a further 5% (2/40). This resulted in upstaging for 20% (8 out of 40) of patients (15% because of BT, 2.5% because of ulceration, and 2.5% because of BT and ulceration). The upstaging effect was incremental, with approximately 5% of patients upstaged with each additional 100 μm interval (up to 400 μm). Incipient ulceration and epidermal consumption were infrequent (10% of cases); however, when present, ulceration was subsequently observed in half of cases. We encountered no cases where microsatellitosis was detected at deeper levels.

**Conclusion:**

The performance of additional tissue levels is a simple and inexpensive procedure that can improve the accuracy of staging for patients with thin (pT1) primary cutaneous melanomas. It may be pertinent for pathologists to consider additional levels for thin melanomas when a BT measurement is close to a staging threshold (e.g., within 0.1–0.3 mm for pT1a vs. pT1b, or pT1b vs. pT2a), or when incipient ulceration is encountered.

## 1 Introduction

Breslow thickness (BT) is the most important prognostic parameter in the assessment of primary cutaneous melanoma [1, 2]. Taken together, BT and ulceration determine the pathological T (pT) stage of melanoma in the eighth edition of the AJCC Cancer Staging Manual [2]. In addition, the presence of microsatellites has prognostic significance comparable to nodal basin involvement and is now incorporated into pathological N stage [2]; in its presence, melanoma is categorized as Stage II disease.

As originally emphasized by Breslow in 1977, melanoma tumor thickness can be spatially heterogeneous [3]. Most macroscopic handling protocols, therefore, advocate that primary melanomas are submitted for histopathologic examination in their entirety, a process that results in tissue slices of approximately 2–3 mm thickness [4, 5]. Depending on the number and depth of levels examined from such slices, a handful of studies have shown that BT and other prognostic factors can vary [6, 7, 8, 9, 10, 11].

There is no standardized recommendation for the number or depth of levels a pathologist should examine microscopically when evaluating melanoma [4, 5]. It is the authors' experience working in several high‐volume melanoma referral centers that the number of levels evaluated varies considerably across and within institutions. A large survey of US dermatopathology practices also found inconsistency [12]. Standardization of the number and depth of levels has not been addressed for the multi‐institution data sets used by AJCC [2].

Although staging systems derived from large patient data sets can account for population variability, many of an individual patient's melanoma prognostic parameters are reported in a binary fashion and directly determine management. Given that vertical tumor thickness alters the primary T stage category in increments of 0.1 mm, foci of ulceration can appear “incipient” at the edges, and microsatellites are often submillimeter, there is value in understanding the degree of variability that may occur when a number of tissue levels are examined histopathologically [2, 13, 14, 15].

Of the few studies that have formally addressed this issue, most have been inconsistent in the distances of the intervals examined [6, 7, 8, 9, 10, 11]. Most previous studies have evaluated only a single parameter [6, 7, 8, 9, 10, 11].

We evaluated the variability in prognostic parameters that occurred when increased numbers of tissue levels taken at regular intervals were examined histopathologically for the primary prognostic determinants of the AJCC stage, namely BT, ulceration, and microsatellites. We also sought to determine whether the assessment of multiple tissue levels beyond the initial hematoxylin and eosin (H&E) staining is warranted, and if so, how many levels, and at what depth, would optimize assessment. To the best of our knowledge, this is the first study to consider variability when multiple histopathologic parameters are evaluated in multiple histopathological tissue sections taken at regular intervals in a single study.

## 2 Methods

We studied 40 consecutive cases with an unequivocal diagnosis of primary cutaneous (nonacral) melanoma treated at a major melanoma referral center (Peter McCallum Cancer Center). Only patients with invasive melanomas were included in this study. Melanomas that were already maximally staged as pT4b with microsatellites and those with clinically evident lymph node metastases were excluded.

Specimens included all complete excision biopsies, punch biopsies (excisional or partial), shave biopsies (excisional or partial), and incisional biopsies, for which the primary purpose of diagnosing and staging melanoma. Wide local excisions of previously biopsied invasive melanomas were excluded. Very small biopsies with a significant risk of tissue cutting out on levels were also excluded.

All melanomas were sliced macroscopically at 2–3 mm intervals, submitted in their entirety for pathological examination, and routinely processed into formalin‐fixed, paraffin‐embedded blocks. After a diagnosis of unequivocal melanoma was made on an initial H&E‐stained section, four additional sections at 100 μm intervals were cut and stained.

In cases where invasive melanoma was present in multiple blocks, additional levels were performed and evaluated on all relevant blocks, and the highest parameters were recorded.

Age, sex, site, melanoma subtype, and biopsy modality were recorded for each patient. The following histological parameters were assessed for each H&E slide: measurement of BT (to the nearest 0.1 mm), ulceration (present, absent, or incipient), AJCC pT stage, microsatellites, lymphovascular invasion (LVI), tumor‐infiltrating lymphocytes (TILs, examined according to ICCR recommendations), and intermediate or late regression. The mitotic rate was performed on only one level in an area deemed to be the tumor hotspot. BT was measured using an ocular graticule, calibrated for 0.1 mm increments. Microsatellites were defined according to the AJCC staging manual (eighth edition), where consensus pathological opinion was that the tumor represented a spatially distinct tumor not separated by fibrosis or other abnormal tissue reactions. The AJCC stage was compared between the first level and any subsequent upgrade.

To account for intra‐ and inter‐observer variability, cases were evaluated with initial and repeat evaluations at least 3 months apart (L.J.), with a second pathologist validation assessment (J.G.).

## 3 Results

Forty patients were analyzed. The clinicopathological parameters of the patients are outlined in Table 1. The majority of patients were male (65%, 26 out of 40), and the age range was 44–92 years (mean, 72 years; median, 75 years). Most cases were diagnostic excision biopsies (34 out of 40, 85%) and most were located on the head and neck (17 out of 40, 42.5%). Superficial spreading melanoma was the most common subtype (20 out of 40; 50%).

**TABLE 1:** Clinicopathological characteristics of the studied 40 cases.

Characteristic	No. of cases (*n* = 40)	%
Age (mean = 72, median = 75)		
< 40	0	0%
40–49	1	2.5%
50–59	5	12.5%
60–69	10	25%
70–79	10	25%
80+	14	35%
Gender		
M	26	65%
F	14	35%
Biopsy type		
Excision biopsy	34	85%
Shave	2	5%
Punch biopsy	2	5%
Incisional	2	5%
Subtype		
SSM	20	50%
LMM	12	30%
NM	4	10%
DM	2	5%
Not able to be determined	1	2.5%
LMM with DM	1	2.5%
Site group		
Head and neck	17	42.5%
Upper limb	10	25%
Trunk	8	20%
Lower limb	5	12.5%
Mitotic rate (per mm^2^)		
Mean	2.8	
Std. deviation	4.39	
Range	0–21	
Tumor infiltrating lymphocytes		
Non‐brisk	14	35%
Absent	9	22.5%
Brisk—ICCR pattern 1	10	25%
Brisk—ICCR pattern 2	7	18%
Intermediate or late regression		
Absent	26	65%
Present—associated	9	22.5%
Present—un‐associated	5	12.5%

### 3.1 Breslow Thickness

**FIGURE 1:**
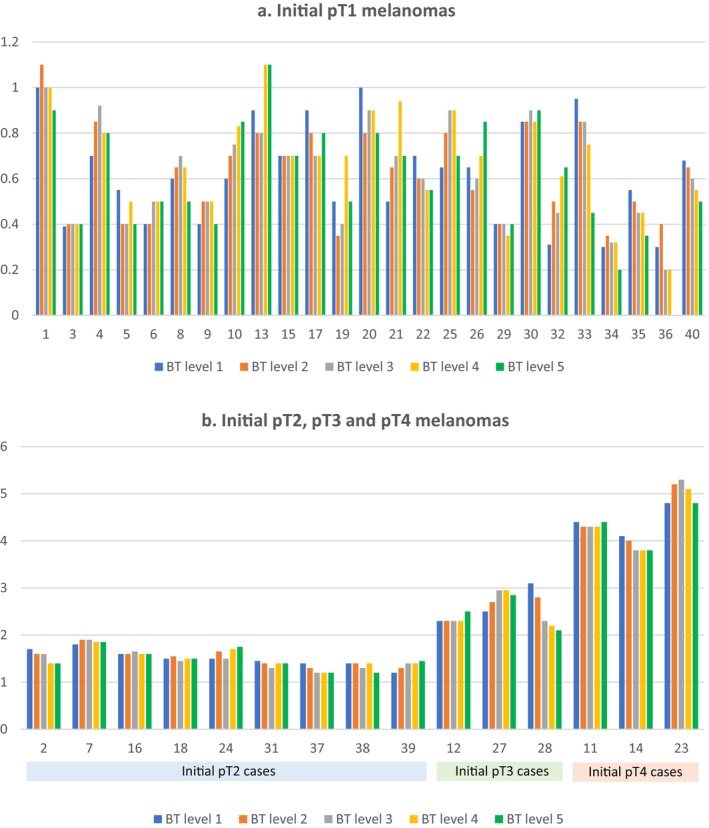
Variability of Breslow thicknesses (BT) across five sequential levels at 100 μm depth intervals, for each of the 40 cases, grouped as (a) initial pT1 melanomas and (b) initial pT2, pT3 and pT4 melanomas (*y* axis = BT in mm; *x* axis = case numbers, batches).

**FIGURE 2:**
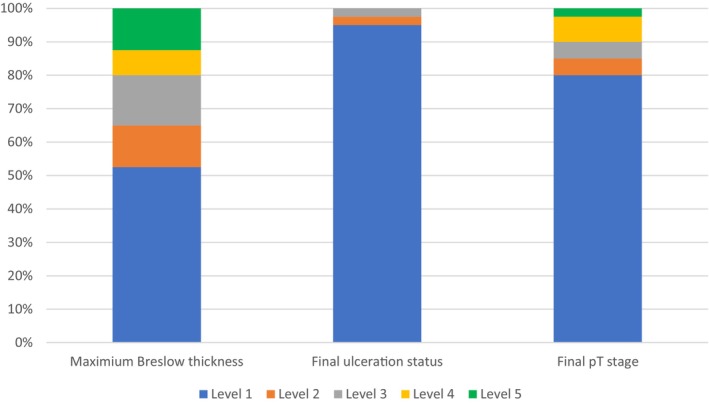
Level at which maximal Breslow thickness, final ulceration status, and final pathological T (pT) stage are determined, as a proportion of all cases (*n* = 40).

**FIGURE 3:**
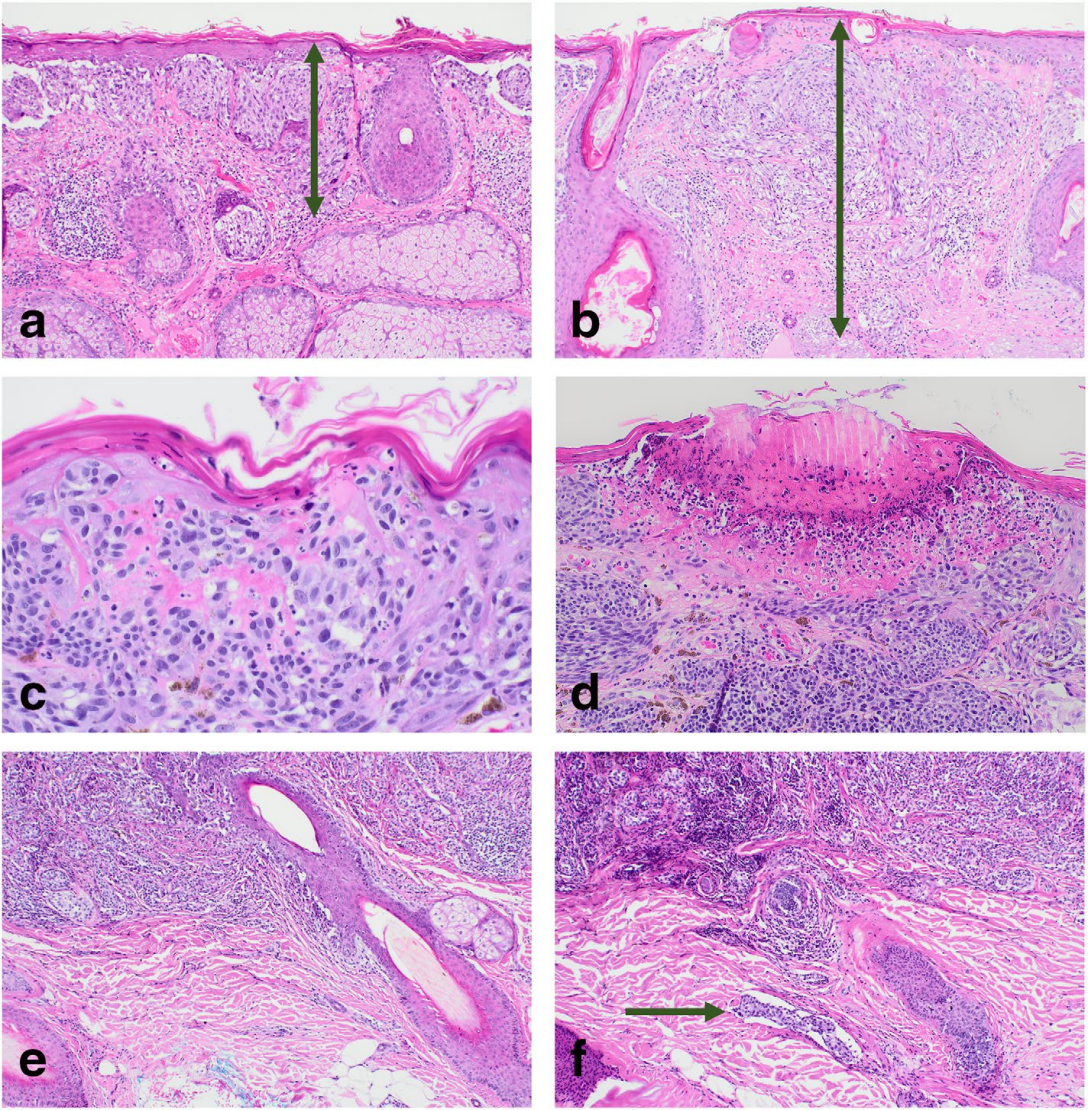
Microscopic images of three case examples. (a H&E ×100) and (b H&E ×100) Case 21:82M forehead lentigo maligna melanoma (LMM), Breslow thickness (BT) 0.5 mm on Level 1 (a) and 0.9 mm on Level 4 (b), resulting in upstaging from pT1a to pT1b. (c H&E ×400) and (d H&E ×200) Case 39:82M, LMM on arm, BT 1.5 mm, showing incipient ulceration with fibrin and epidermal consumption on Level 2 (c), and ulceration at the next 100 μm interval on Level 3 (d). This resulted in the upstaging from pT2a to pT2b. (e H&E ×100) and (f H&E ×100) Case 31:79M, LMM on scalp, BT 1.4 mm, showing no LVI on the first three levels (g), and LVI on level 4 (h). (This did not result in a change in pT.)

On the initial level, the mean BT of all cases was 1.3 mm (0.3–4.8 mm, median 0.9, Figure 1a,b). After review of additional levels, there was a change in maximum BT in 47.5% (19 out of 40) of the cases (Table 2, Figures 2 and 3a,b). When there was an increase in BT from the initial levels, the change was an average of 0.11 mm (range 0.1–0.5 mm).

**TABLE 2:** Changes in Breslow thickness (BT), ulceration, and pathological T stage.

Characteristic	No. of cases (*n* = 40)	%
Change in BT		
No	21	52.5%
Yes	19	47.5%
Maximum BT achieved at which level?		
Level 1	21	52.5%
Level 2	5	12.5%
Level 3	6	15%
Level 4	3	7.5%
Level 5	5	12.5%
Change in ulceration status		
No	38	95%
Yes	2	5%
Final ulceration status achieved at which level?		
Level 1	38	95%
Level 2	1	2.5%
Level 3	1	2.5%
Level 4	0	0%
Level 5	0	0%
Change in pathological T (pT) stage?		
No	32	80%
Yes	8	20%
Final pT achieved at which level?		
Level 1	32	80%
Level 2	2	5%
Level 3	2	5%
Level 4	3	7.5%
Level 5	1	2.5%

Of these cases, the final BT was achieved at Level 2 in five cases (5 out of 19, 26%), Level 3 in six cases (6 out of 19, 32%), Level 4 in three cases (3 out of 19, 16%), and Level 5 in five cases (5 out of 19, 26%).

### 3.2 Ulceration

Ulceration was present in 7.5% (3 out of 40) of cases in the initial slide review. In addition, four cases (10%) had features of incipient ulceration, and the additional levels confirmed true ulceration in two of these cases (5%, Table 2, Figure 2). In one case, the final ulceration status was observed on Level 2 (Figure 3c,d), and the other on Level 3.

### 3.3 pT Stage

In the majority of cases (32 out of 40, 80%), the pathological stage did not change with levels beyond Level 1. However, eight cases (8 out of 40, 20%) were upstaged after evaluating additional levels (Tables 3 and 4). This included five cases in which pT1a was changed to pT1b, one case in which pT1b was changed to pT2a, one case in which pT1b was changed to pT2b, and one case in which pT2a was changed to pT2b. The changes in parameters, and the respective levels, are outlined in Table 4.

**TABLE 3:** Distribution of pathological T (pT) stage observation at respective batch levels, No. of cases (%).

Level	pT1a	pT1b	pT2a	pT2b	pT3a	pT3b	pT4a	pT4b
1	19 (47.5)	6 (15)	9 (22.5)	0 (0)	2 (5)	1 (2.5)	1 (2.5)	2 (5)
2	18 (45)	6 (15)	10 (25)	0 (0)	2 (5)	1 (2.5)	1 (2.5)	2 (5)
3	17 (42.5)	7 (17.5)	9 (22.5)	1 (2.5)	2 (5)	1 (2.5)	1 (2.5)	2 (5)
4	15 (37.5)	8 (20)	9 (22.5)	2 (5)	2 (5)	1 (2.5)	1 (2.5)	2 (5)
5	14 (35)	9 (22.5)	9 (22.5)	2 (5)	2 (5)	1 (2.5)	1 (2.5)	2 (5)

**TABLE 4:** Features of the eight cases where there was a change in pathological T (pT) stage after evaluation of levels.

Case number	pT stage at Level 1	Breslow thickness at Level 1	Ulceration status at Level 1	Final pT status	Final BT (level identified)	Final ulceration status (level identified)
21	pT1a	0.5 mm	Absent	pT1b	0.9 mm (Level 4)	Absent
10	pT1a	0.6 mm	Absent	pT1b	0.9 mm (Level 4)	Absent
4	pT1a	0.7 mm	Absent	pT1b	0.9 mm (Level 2)	Absent
25	pT1a	0.7 mm	Absent	pT1b	0.9 mm (Level 3)	Absent
26	pT1a	0.7 mm	Absent	pT1b	0.9 mm (Level 5)	Absent
1	pT1b	1 mm	Absent	pT2a	1.1 mm (Level 2)	Absent
13	pT1b	0.9 mm	Incipient	pT2b	1.2 mm (Level 4)	Present (Level 2)
39	pT2a	1.2 mm	Incipient	pT2b	1.5 mm (Level 3)	Present (Level 3)

Changes in BT, ulceration, and final pT status were not significantly associated with sex, melanoma subtype, site, TILs, or intermediate and late regression.

### 3.4 Microsatellitosis

Microsatellitosis was identified in two cases (2 out of 40, 5%), one for T2b melanoma and one for T4a melanoma, but this was present at all levels examined. There were no cases in which convincing microsatellitosis was identified by studying the additional levels.

We noted that for one case (2.5%), a pT4b melanoma, we found a small deposit of melanoma associated with a lymphoid aggregate in the subcutis at Level 2, which appeared to be physically separated from the main melanoma by a thrombosed blood vessel, the nature and significance of which were difficult to classify, but we were not convinced that this constituted a true microsatellite.

### 3.5 Lymphovascular Invasion

No cases of LVI were identified in the first‐level evaluation. While it did not affect the pathological staging, in one case (2.5%), a pT2a melanoma, we identified a small lymphovascular channel tumor deposit that turned up on Level 4 (Figure 3e,f).

## 4 Discussion

In an era where nonanatomic factors are increasingly relevant to precision medicine, the assessment of key histopathologic parameters of primary melanoma remains one of the most powerful tests to determine a patient's prognosis and guide management decisions.

The 8th edition of the AJCC Cancer Staging Manual incorporates three key anatomical factors of primary melanoma (BT, ulceration, and microsatellites) into pathological staging [2]. Mitotic rate and LVI remain important independent prognostic markers and are utilized in prognostic nomograms, but are not currently incorporated into the formal AJCC stage categories [16, 17].

Several studies have shown that many features of melanoma are subject to variation based on the profile of the tumor that is examined in a particular plane of section [6, 7, 8, 9, 10, 11]. This phenomenon is not unique to melanoma and has been documented in a range of tissue types and tumor pathologies [18, 19, 20].

Being a highly prevalent disease with major global health and economic burdens, maximizing the information from H&E assessment of melanoma is desirable because H&E slides are cheaper and easier to produce than ancillary techniques, such as immunohistochemistry (IHC) and molecular analysis. Melanoma research that considers equity for underresourced and pressured health systems is valuable.

### 4.1 Breslow Thickness

We have shown that the BT is influenced by the number of tissue levels examined. We found additional levels resulted in an increase in BT of at least 0.1 mm for 47.5% (19 out of 40) of cases, with an average increase of 0.1 mm (range 0.1–0.5 mm). This resulted in upstaging in seven cases (17.5%), all of which were initially staged as pT1.

Our findings are in line with a limited number of H&E‐only studies that have encountered a similar upstaging effect in 9%–18.5% of cases [6, 7, 10, 11]. In one series of 54 melanomas, this effect was significant when the first four levels were examined, with no additional significant benefit between Levels 5 and 10 [6]. This is also concordant with our findings, where we found that 80%, 85%, 90%, and 98% of cases will be maximally staged at Levels 1, 2, 3, and 4 (of a total of five levels), respectively (Table 2).

Two studies compared the measurement of BT between H&E and IHC stains (S100, melanA, HMB45 ± Sox10) [8, 9]. In one study, 29 out of 41 invasive melanomas (70%) showed an increase in BT of at least 0.1 mm after quad‐panel IHC stains compared to the H&E slide, which led to a change in pT stage in 11 cases (11 out of 41, 26%) [8]. In another study, greater tumor thickness was observed in 27 lesions (27 out of 36, 75%) after a combination of three IHC stains (S100, melanA, HMB45) compared to the initial H&E staining, resulting in a one‐step increase in the pT score for 22% of lesions [9]. In both studies, most cases with significant changes were thin (pT1) melanomas. Both groups concluded that IHC staining led to a superior evaluation of BT compared to H&E staining. However, since their findings are similar to ours, a clinically significant change in BT is likely to be, at least in part, a function of the additional levels being cut and examined rather than the IHC stain per se. Considering the significant cost and resources required to generate IHC stains compared with H&E staining, this is a judicious point to note. However, we recognize that IHC stains serve other functions in the workup of melanoma cases.

### 4.2 Ulceration

The importance of ulceration as a poor prognostic factor has been well documented [21, 22, 23]. In our study, we found that in the absence of deeper levels, ulceration would have been missed in two cases (5%). This is concordant with Dyson et al. who found that ulceration was detected only by examining deeper levels in 3% of the cases [7].

Several investigators have also shown that epidermal consumption and incipient ulceration have similar prognostic significance to true ulceration [7, 14]. Dyson et al. and Paver et al. showed that incipient ulceration lay adjacent to true ulcerations in additional sections. These observations suggest that epidermal consumption, incipient ulceration, and true ulceration are on a spectrum and that patients may be under‐staged if only the former tissue patterns are profiled in tissue sections [14, 15]. We identified only four patients whose melanomas had epidermal changes consistent with consumption or incipient ulceration, two of which revealed true ulceration at the subsequent level. This suggests that epidermal consumption and incipient ulceration are uncommon findings; however, their recognition should trigger a pathologist to examine further.

### 4.3 Microsatellitosis

To the best of our knowledge, no study has specifically evaluated how tissue levels influence the detection of microsatellites and LVI. We did not find any cases of microsatellitosis picked up at deeper levels, suggesting that the performance of levels solely for this purpose is likely to be of low yield.

## 5 Limitations

Our study had several limitations. By including partial biopsies (punch, shave, and incisional biopsies, which accounted for 15% of our cohort), prognostic parameters cannot always be fully assessed. However, we included them to be reflective of wider clinical practice because it is important for pathologists to report all parameters regardless of the specimen type.

We did not include wide local excisions and recognized that these specimens will often determine a patient's final BT. However, we specifically excluded these cases so that biopsy site changes did not contaminate the measurement of the prognostic parameters.

We tried to address a limitation of prior literature, in which most studies did not specify the depth at which tissue levels were cut [6, 8, 9]. The lack of standardization in prior studies means that it is difficult to determine optimum sampling standards. In the only available study that attempted to standardize interval distance, Patrick et al. produced 10 slides at 40 μm intervals and found a ceiling effect at around five to seven levels, equating to approximately 200 μm of tissue examined [11]. In contrast, Dyson et al. evaluated more variable amounts of tissue, using intervals of 50–100 μm between the initial two slides (depending on specimen size), and 300–500 μm between five additional slides, to examine through an entire block [7]. By their own admission, they did not set out to establish a standard for optimal tissue sampling. However, sampling through an entire block presents practical and ethical problems, particularly in an era when primary tissue may be an invaluable resource for molecular testing to guide targeted and other modern treatments.

To address this gap in the literature and improve its application to wider laboratory practice, we chose five batches at 100 μm intervals (a total of 400 μm between Levels 1 and 5), which represents approximately 20% of a 2‐mm tissue slice. This is an interval that is relatively common across routine laboratory practice, but also balances the need to ensure that lesional tissue is not exhausted from the block for potential ancillary testing. We recognize that intervals of 50 μm may be more appropriate for very small specimens, and the information garnered from our study should assist pathologists in determining how much tissue should be examined in such cases.

## 6 Clinical and Staging Value of Examining Multiple Tissue Levels

Understanding the variability of information that can be obtained from additional tissue levels is useful for pathologists who have (a) an obligation to use laboratory resources judiciously and (b) a responsibility to balance tissue evaluation with the need to preserve tissue for ancillary studies, now and in the future.

Our study demonstrates that when compared to the initial H&E section, examination of additional tissue levels in 100 μm increments resulted in (a) an increase in the BT in 47.5% of cases, and (b) detection of ulceration in a further 5% of cases. This correlated with upstaging of patients' pT stage in 20% of cases (15% because of BT alone, 2.5% because of ulceration alone, and 2.5% because of BT and ulceration). We found no relationship between changes in parameters and melanoma subtype, site, age, mitotic rate, initial BT, TIL, or regression.

There is an incremental effect, such that 80% of cases of melanoma were accurately pathologically staged at the initial level, with roughly another 5% of patients being upstaged with each additional 100 μm interval examined (up to 400 μm). We found that only cases initially staged as pT1 were eventually upstaged because of increased BT; the average increase in BT was 0.11 mm. This suggests that it is pertinent for pathologists to consider performing additional levels on thin (pT1) melanomas when a BT assessment is within 0.1–0.3 mm of a threshold measurement (i.e., pT1a vs. pT1b or pT1b vs. pT2a).

Incipient ulceration and epidermal consumption are rare findings; however, when present, ulceration was subsequently observed in half of the cases. Incipient ulceration may, therefore, serve as a clue for true ulceration, and it is worth a pathologist pursuing additional levels.

Additional levels only rarely identify other prognostic parameters such as LVI and microsatellitosis.

Our study adds to several other studies that have shown changes in BT and staging using immunohistochemical staining. However, our observations suggest that the improved evaluation may be a function of leveling the tissue rather than the stain per se. IHC does have other roles, and pathologists should incorporate immunohistochemical studies based on their judgment.

Importantly, the upstaging effect observed in our study may help explain why a small percentage of patients with thin lesions (≤ 1.0 mm) have unexpectedly poor prognoses [24, 25]. Given this potential clinical impact, our findings will undoubtedly raise questions for many pathologists and laboratories regarding how and if they should change their practice.

In answering this question, we emphasize the importance of the pathologist's judgment and discretion. While we recommend levels for thin pT1 lesions where the measurement is within 0.1–0.3 mm of the next T category, or when “incipient” ulceration is identified, we recognize that a pathologist's decision to examine additional tissue must consider multiple factors. These include the degree of diagnostic certainty, biopsy type and size, overall size of the lesion, tissue patterns such as regression, need for IHC, and rationalization of the tissue for molecular testing. In our study, we specifically kept our cohort to unequivocal melanomas and cases where there was no significant risk of tumor cutting out; however, this does not account for all real‐life encounters with melanomas.

It is hoped that our findings will alert pathologists to instances where additional tissue‐level examination may be judged as most beneficial, rather than mandate a radical change in practice. Importantly, we caution laboratories performing multiple levels upfront in all cases of melanocytic lesions. Doing so could risk the loss of precious tissue and is likely to lead to injudicious use of resources for many patients. However, we hope that our findings will provide evidence for the degree of variability in prognostic parameters, of which many pathologists will already be aware anecdotally, and allow them to make informed decisions regarding the benefits and potential costs of cutting through further tissue.

In conclusion, the performance of additional tissue levels is a simple and inexpensive test that can provide valuable prognostic information for patients with thin (pT1) primary cutaneous melanomas. Its value lies mostly in the evaluation of pT1 melanomas with an initial BT that is within 0.1–0.3 mm of the threshold for the next T category, and in the rare instance that incipient ulceration is identified. Pathologists should use discretion and judge cases on an individualized basis.

## Author Contributions

L.A.J. conceived of the project. L.A.J. and R.A.S. designed the study and methodology. L.A.J. identified the patient cohort, collected all data, and analyzed the data. J.P.G. performed validation analysis on the collected data. All authors discussed the results and contributed to the final manuscript.

## Disclosure

The authors have nothing to report.

## Ethics Statement

Ethics approval and waiver use of medical information has been granted through independent ethics committees and research boards (reference Peter Mac No: 24/73R, HREC Reference: HREC/108528/PMCC).

## Conflicts of Interest

Richard A. Scolyer has received fees for professional services from SkylineDx BV, IO Biotech ApS, MetaOptima Technology Inc., F. Hoffmann‐La Roche Ltd., Evaxion, Provectus Biopharmaceuticals Australia, Qbiotics, Novartis, Merck Sharp & Dohme, NeraCare, AMGEN Inc., Bristol‐Myers Squibb, Myriad Genetics, and GlaxoSmithKline. The other authors declare no conflicts of interest.

## Data Availability

Research data supporting this publication are available on request from the author.
